# A Quantitative Method to Measure Low Levels of ROS in Nonphagocytic Cells by Using a Chemiluminescent Imaging System

**DOI:** 10.1155/2019/1754593

**Published:** 2019-06-11

**Authors:** Jun-Sub Kim, Kyuho Jeong, James M. Murphy, Yelitza A. R. Rodriguez, Ssang-Taek Steve Lim

**Affiliations:** ^1^Department of Biochemistry and Molecular Biology, College of Medicine, University of South Alabama, Mobile, AL 36688, USA; ^2^Department of Biotechnology, Korea National University of Transportation, Jeungpyeong 27909, Republic of Korea

## Abstract

Chemiluminescence (CL) is one of the most useful methods for detecting reactive oxygen species (ROS). Although fluorescence dyes or genetically encoded biosensors have been developed, CL is still used due to its high sensitivity, ease of use, and low cost. While initially established and used to measure high levels of ROS in phagocytic cells, CL assays are not ideal for measuring low levels of ROS. Here, we developed a newly modified CL assay using a chemiluminescent imaging system for measuring low concentrations of ROS in nonphagocytic cells. We found that dissolving luminol in NaOH, rather than DMSO, increased the H_2_O_2_-induced CL signal and that the addition of 4-iodophenylboronic acid (4IPBA) further increased CL intensity. Our new system also increased the rate and intensity of the CL signal in phorbol 12-myristate 13-acetate- (PMA-) treated HT-29 colon cancer cells compared to those in luminol only. We were able to quantify ROS levels from both cells and media in parallel using an H_2_O_2_ standard. A significant benefit to our system is that we can easily measure stimulus-induced ROS formation in a real-time manner and also investigate intracellular signaling pathways from a single sample simultaneously. We found that PMA induced tyrosine phosphorylation of protein tyrosine kinases (PTKs), such as focal adhesion kinase (FAK), protein tyrosine kinase 2 (Pyk2), and Src, and increased actin stress fiber formation in a ROS-dependent manner. Interestingly, treatment with either N-acetyl-L-cysteine (NAC) or diphenyleneiodonium (DPI) reduced the PMA-stimulated phosphorylation of these PTKs, implicating a potential role in cellular ROS signaling. Thus, our newly optimized CL assay using 4IPBA and a chemiluminescent imaging method provides a simple, real-time, and low-cost method for the quantification of low levels of ROS.

## 1. Introduction

Early research on reactive oxygen species (ROS) was primarily focused in the field of innate immunity; in particular, NADPH oxidase 2- (Nox2-) mediated ROS generation during phagocytosis by phagocytes has been extensively studied [1]. Since new Nox genes were discovered, studies on ROS have extended to nonphagocytic cells and various disease models including cancer [1, 2]. Accordingly, ROS have been shown to be important as both an oxidative source and a secondary messenger within cellular signaling events [3]. ROS promote growth and survival at low physiological intracellular levels (0.001-0.7 *μ*M), whereas higher levels (20-200 *μ*M) can induce growth arrest, and levels above that eventually cause cell death [3, 4]. Several ROS-sensitive signaling pathways have been found to be elevated in many types of cancers [5], especially signaling pathways including protein tyrosine kinases (PTKs) connected to epithelial-mesenchymal transition, the differentiation process during cancer cell metastasis requiring degradation of the extracellular matrix, high cell mobility, and low focal adhesion dynamics [6].

Although development of techniques and materials for detecting and quantifying ROS has been challenging, numerous methods based on absorption, fluorescence spectroscopy, or chemiluminescence (CL) have been developed [7]. The most commonly used methods are those based on CL, in which ROS react with various probes, such as lucigenin, L-012, or luminol, to generate light. While these probes react with superoxide to generate light, luminol can also react with H_2_O_2_, making it a common probe for CL systems [7]. Luminol-based CL has primarily been used for neutrophil, macrophage, or Nox system-derived ROS [8–10] and is a very simple, highly sensitive, and cost-effective method compared to others and is therefore widely applied in high-throughput systems [11]. However, it is still difficult to quantify low levels of stimulus-induced ROS generation in nonphagocytes.

Luminol-based CL has primarily been used for western blotting rather than measuring cell-derived ROS. Several studies have used various enhancers for western blotting, termed enhanced chemiluminescence (ECL), to further intensify CL signal [12–14]. We focused on using 4-iodophenylboronic acid (4IPBA) as an enhancer for luminol-based CL detection of low concentrations of ROS in nonimmune cells by using a chemiluminescent imaging system. Additionally, we attempted to develop a system that allowed us to measure ROS released by cells into media and use cell lysates to investigate intracellular signaling pathways to find a new marker of ROS generation. Oxidation of proteins, lipids, and nucleic acids, which cause serious cellular damage or cell death, has been used as markers for high levels of ROS [15]. While low concentrations of ROS are also involved in various signaling pathways, there is lack of a clear marker as an indicator of low ROS levels [16]. Interestingly, ROS have been linked to activation of several PTKs, such as focal adhesion kinase (FAK), proline-rich tyrosine kinase 2 (Pyk2), and Src [17–21]. Here, we have established a new chemiluminescent imaging method to measure micromolar (*μ*M) levels of ROS in nonimmune cells and suggest that tyrosine phosphorylation of PTKs could be used as markers of ROS generation.

## 2. Materials and Methods

### 2.1. Cells and Reagents

HT-29 human colon carcinoma was obtained from ATCC and maintained in DMEM containing 10% FBS (Omega Scientific), 1 mM sodium pyruvate, 0.1 mM nonessential amino acids, and 100 units/ml penicillin and 100 *μ*g/ml streptomycin. Diphenyliodonium (DPI), N-acetyl-L-cysteine (NAC), phorbol 12-myristate 13-acetate (PMA), horseradish peroxidase (HRP), luminol, H_2_O_2_, and 4-iodophenylboronic acid (4IPBA) were purchased from Sigma-Aldrich. Superoxide dismutase (SOD) was purchased from Abnova, and catalase (CAT) was purchased from MP Biomedicals.

FAK (Millipore), pY397 FAK (Invitrogen), pY402 Pyk2 (Cell Signaling), pY416 Src (Invitrogen), GAPDH (Millipore) antibody, and Alexa 488 phalloidin (Invitrogen) were obtained.

### 2.2. Chemiluminescence Assay

Chemiluminescence (CL) was measured by luminol-amplified luminescence, and optimized CL buffer compositions and storage conditions are summarized in Tables 1 and 2. All CL experiments were carried out in 200 *μ*l total volume in black-walled glass bottom 96-well plates, and luminescence was quantified using a luminometer (Synergy Microplate Reader, BioTek). To measure cellular ROS, overnight serum-starved HT-29 cells were trypsinized and suspended in Hank's Balanced Salt Solution (HBSS) and then 2 × 10^5^ cells were mixed with a CL buffer. For released ROS measurement, overnight serum-starved HT-29 cells (1.2 × 10^6^) were incubated in 1 ml Opti-MEM. Cells were then treated with or without NAC (10 mM) or DPI (10 *μ*M) for 1 h prior to stimulation with PMA (200 nM) for 30 min. HBSS-suspended cells or cleared conditioned media were used for the CL assay by using a ChemiDoc MP Imaging System (Bio-Rad) in SAM (signal accumulation mode, exposure time 30 sec, total duration for 300 sec). Density of CL was quantitated using Image Lab Software (Bio-Rad).

### 2.3. Immunoblotting

HT-29 cells were serum-starved overnight and then treated with or without NAC (10 mM) or DPI (10 *μ*M) for 1 h prior to PMA (200 nM) stimulation. Cells were lysed in 1% Triton X-100 lysis buffer, and clarified lysates were run on 4-12% NuPAGE Tris-Bis gels (Life Technologies). Protein was transferred to PVDF membranes, blocked with 3% BSA, and incubated overnight with primary antibodies at 4°C. Membranes were washed and incubated with HRP-conjugated secondary antibodies, and proteins were then visualized using ECL on the ChemiDoc MP Imaging System.

### 2.4. Immunofluorescence Staining

HT-29 cells were seeded on 0.1% gelatin-coated coverslips and treated as described above. Cells were then fixed with 4% PFA and permeabilized and blocked with 0.1% Triton X-100, 3% BSA, and 1% goat serum. Coverslips were incubated with primary antibodies overnight at 4°C and were then washed with PBS and incubated with secondary antibodies for 1 h at room temperature. Coverslips were mounted with Fluoromount-G (SouthernBiotech), and images were acquired with a Nikon A1R confocal microscope.

### 2.5. Statistical Analysis

Statistical significance was evaluated by *t*-test or ANOVA, and *p* < 0.05 was considered to be statistically significant. Analyses were performed using GraphPad Prism.

## 3. Results

### 3.1. Optimization of the Luminol-Based Chemiluminescence Assay Using 4IPBA to Detect Low Levels of ROS

**Figure 1:**
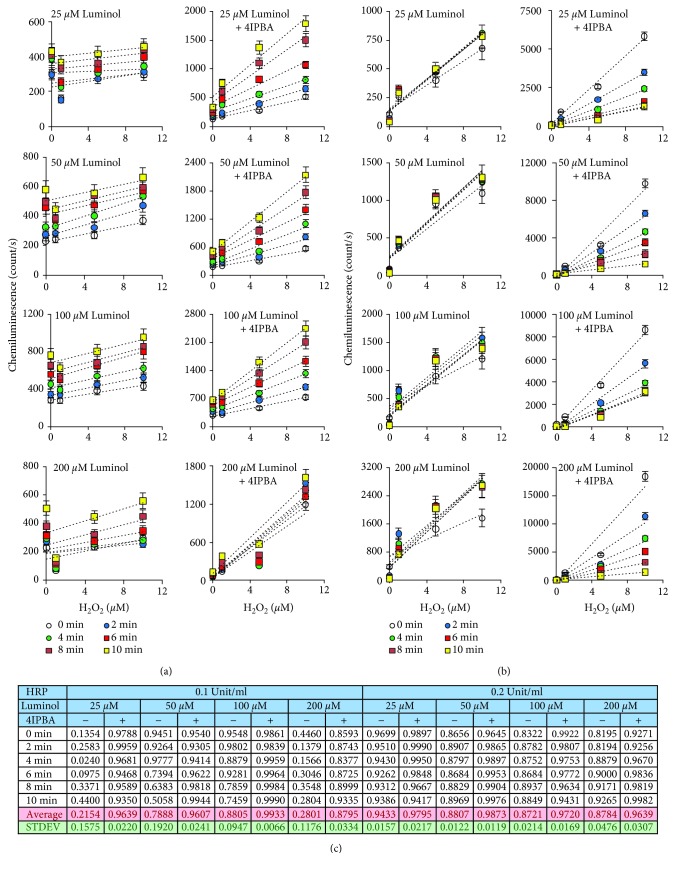
Optimization of a luminol-based chemiluminescence assay using a luminometer. Various concentrations of H_2_O_2_ (0, 1, 5, and 10 *μ*M), luminol (25, 50, 100, and 200 *μ*M in NaOH), and 4IPBA (0 or 180 *μ*M) were added to a black-walled glass bottom 96-well plate (see Table 1). Chemiluminescence was measured using a luminometer with either (a) 0.1 unit/ml or (b) 0.2 unit/ml HRP for the indicated times (*n* = 3, ±SD). (c) Shown are *R* ^2^ values for different buffer conditions. *R* ^2^: linear correlation coefficient.

While previous studies have used a fixed concentration of 2.4 mM H_2_O_2_ for enhanced chemiluminescence (ECL) [13], we tested if 4-iodophenylboronic acid (4IPBA) could enhance the CL signal at lower levels of H_2_O_2_ (1-10 *μ*M). By using a luminometer, we optimized CL reaction with two different CL buffers (luminol dissolved in DMSO or NaOH), each of which contained 25-200 *μ*M luminol and 0.1-0.2 units/ml of horseradish peroxidase (HRP) (see Table 1). It is thought that NaOH can promote luminol-based CL reaction by raising the pH and producing NaOCl [8, 22–24]. As expected, luminol dissolved in NaOH produced higher CL signals compared to luminol dissolved in DMSO (Supplemental Figs.  1 and 2). The addition of 180 *μ*M 4IPBA to the reaction dramatically increased CL activity in both CL buffers (Supplemental Figs.  1 and 2). We analyzed a linear correlation coefficient value (*R* ^2^ value) of CL during the first 10 min of luminol in NaOH (Figure 1). The addition of 4IPBA improved the *R* ^2^ value in the presence of both 0.1 and 0.2 units/ml HRP compared to luminol alone (Figure 1). Collectively, we determined two optimal conditions with the highest linear reliability for the CL reaction to detect low levels of H_2_O_2_ to be 0.1 unit/ml HRP, 100 *μ*M luminol, and 180 *μ*M 4IPBA or 0.2 units/ml HRP, 50 *μ*M luminol, and 180 *μ*M 4IPBA (Figure 1(c) and Supplemental Figs.  1 and 2). Complete CL profiles with varying concentrations of luminol, 4IPBA, or HRP are shown in Supplemental Figs.  1 and 2. We also observed that luminol dissolved in NaOH produced higher CL signals by 10- to 50-folds compared to luminol in DMSO (Supplemental Fig.  2). HRP and luminol dose-dependently increased CL activity as well as velocity of CL reaction in both buffers (Supplemental Figs.  1 and 2). CL reactions could detect peaks of the CL curve up to 0.5 units/ml HRP in luminol alone, but up to 0.1 unit/ml HRP in luminol with 4IPBA (Supplemental Fig.  2). In addition, we tested the effect of 4IPBA concentration on CL activity with varying doses of 4IPBA from 0 to 2880 *μ*M. CL activity was dose-dependently increased up to 0-180 *μ*M 4IPBA but decreased with 360–2880 *μ*M 4IPBA (Supplemental Fig.  3).

### 3.2. Development of a Chemiluminescent Imaging Method by Using Luminol-Based Chemiluminescence to Measure Low Levels of ROS

**Figure 2:**
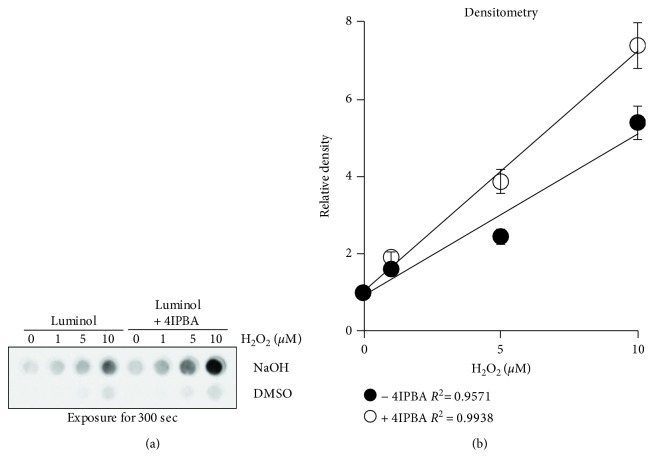
Development of the chemiluminescent imaging method by using luminol-based chemiluminescence to measure low levels of ROS. Various concentrations of H_2_O_2_ (0, 1, 5, and 10 *μ*M) were mixed with luminol (50 *μ*M in DMSO or NaOH) with or without 4IPBA (180 *μ*M) in a black-walled glass bottom 96-well plate. HRP (0.2 U/ml) were added and chemiluminescence was assessed by signal accumulation mode (SAM, every 30 sec for 300 sec) using a chemiluminescent imaging system. (a) Representative chemiluminescent images. (b) Densitometry analyses of chemiluminescent images were plotted (*n* = 3, ±SD). *R* ^2^: linear correlation coefficient.

A previous study measured ROS production in neutrophils using an X-ray film to detect CL from a 96-well plate [24]. Recently, X-ray film systems have been replaced with chemiluminescent imaging systems, which have the benefit of decreased maintenance costs, smaller space, and quicker data acquisition. Thus, we tried to detect CL by using a chemiluminescent imaging system instead of using a luminometer. We were easily able to detect CL using low concentrations of H_2_O_2_ in the 96-well format (Figure 2). Again, luminol dissolved in NaOH had increased signal intensity compared to DMSO, and 4IPBA addition further increased the CL signal (Figure 2). Thus, these results suggest that our new chemiluminescent imaging method with luminol in NaOH produces better CL signals than luminol in DMSO for detecting low levels of ROS. Among all the combinations, the group with luminol, 4IPBA, and NAOH showed the highest CL activity.

### 3.3. Measurement of Low Levels of ROS in PMA-Stimulated HT-29 Colon Carcinoma by Using a New Chemiluminescent Imaging Method

Several studies have shown that phorbol 12-myristate 13-acetate (PMA) can stimulate ROS production in various cell types, including cancer cells in a protein kinase C- (PKC-) and Nox-dependent manner [24, 25]. As the human colon carcinoma HT-29 expresses Nox1 and generates ROS following PMA stimulation [26], we used HT-29 to test if our CL method could detect low concentrations of ROS in HT-29 cells. First, we tested whether our new CL system affects cell viability by using an MTT assay (Supplemental Fig.  4). Although 4IPBA with or without luminol in NaOH did not significantly affect HT-29 cell viability up to 4 h, higher doses of 4IPBA seem to decrease cell viability after 24 h treatment (Supplemental Fig.  4). As the CL assay is usually performed with cells within 60 min, our newly developed method may be suitable for a CL assay without causing cell toxicity during a short period up to 4 h (Supplemental Fig.  4). We also confirmed expression of Nox1 components in HT-29 cells by using RT-qPCR (Supplemental Fig.  5 and Supplemental Table 1).

**Figure 3:**
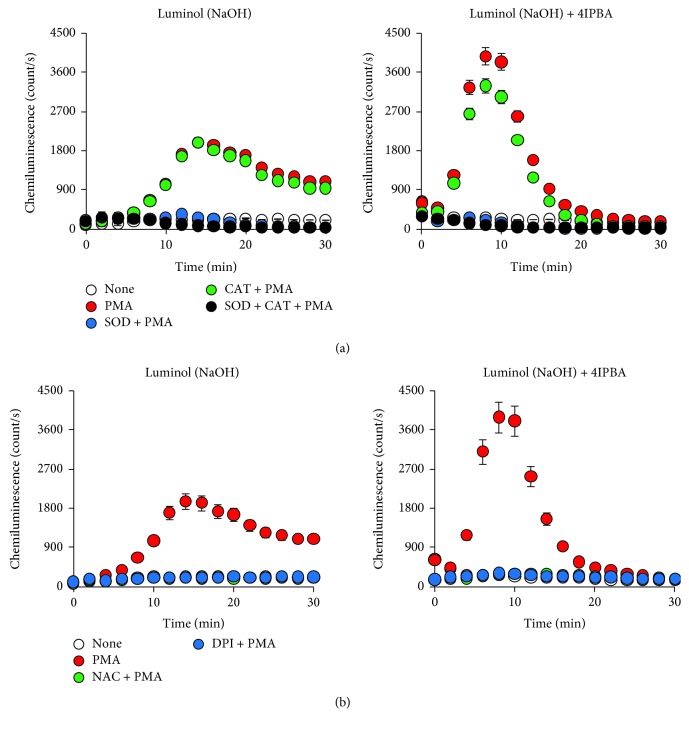
Comparison of chemiluminescence with or without 4IPBA on PMA-induced ROS generation in HT-29 cells. HT-29 cells (2 × 10^5^) were resuspended in luminol (50 *μ*M in NaOH) and HRP (0.2 U/ml) with or without 4IPBA (180 *μ*M) in a black-walled and glass-bottomed 96-well plate. HT-29 cells were then (a) treated with SOD (50 *μ*g/ml) and/or CAT (1.5 kU/ml) or (b) pretreated with NAC (10 mM) or DPI (10 *μ*M) for 1 h. Chemiluminescence was measured using a luminometer for 30 min after PMA (200 nM) stimulation (*n* = 3, ±SD).

Using a luminometer, the CL signal was measured in PMA-treated HT-29 compared to untreated cells (Figure 3). As seen with H_2_O_2_ experiments (Figure 1 and Supplemental Fig.  2), 4IPBA addition further increased the CL intensity and rate compared to luminol alone. Peak signal intensity was achieved at an earlier time point (8 min) with 4IPBA, whereas luminol alone peaked at a later time point (15 min). Additionally, the duration of the signal was reduced with 4IPBA compared to luminol alone (Figure 3). To determine which ROS are being measured, we employed superoxide dismutase (SOD) and catalase (CAT) in PMA-induced HT-29 cells (Figure 3(a)). We observed that while treatment with SOD completely reduced PMA-induced CL intensity, CAT only slightly inhibited the PMA-induced CL signal (Figure 3(a)). These results suggest that most of the CL reaction was mediated by superoxide in PMA-stimulated HT-29 cells. H_2_O_2_ may marginally affected CL activity, but its effects are very low compared to superoxide. Additionally, we pretreated cells with either the ROS scavenger N-acetyl-L-cysteine (NAC) or the Nox inhibitor diphenyleneiodonium chloride (DPI). Treatment with either NAC or DPI blocked PMA-induced CL (Figure 3(b)). These results suggest that PMA induces superoxide formation via Nox1 in HT-29 cells and the new assay system is indeed measuring low levels of ROS in nonphagocytes.

**Figure 4:**
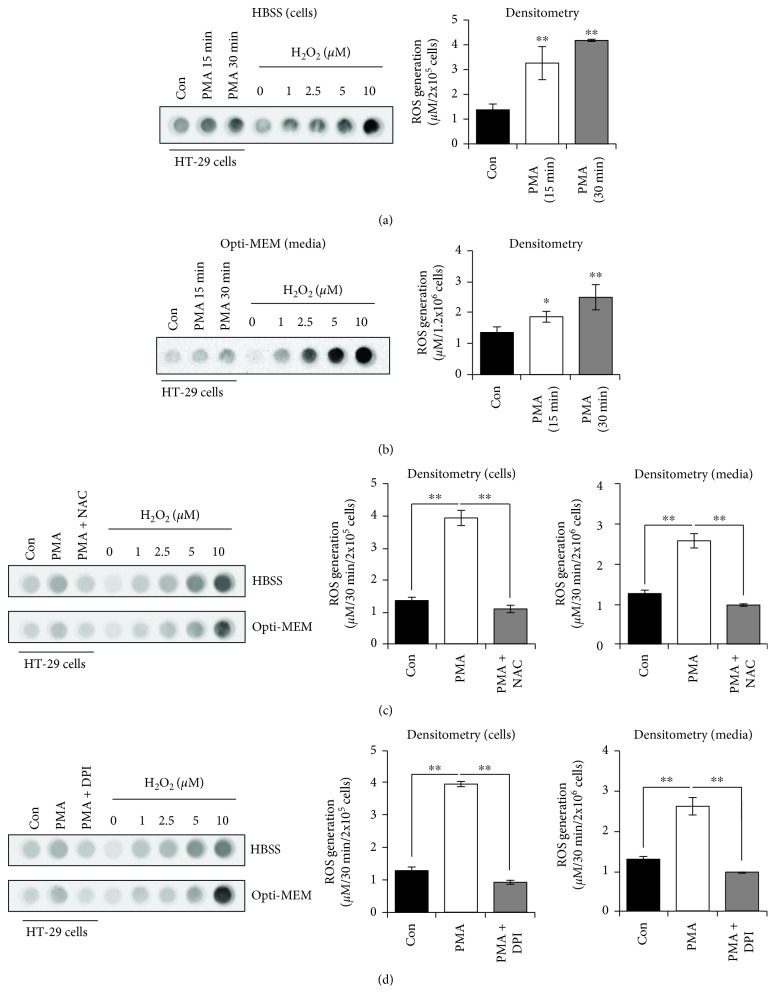
Measurement of low levels of ROS in PMA-stimulated HT-29 cells using the new chemiluminescent imaging method. (a) HT-29 cells (2 × 10^5^) were incubated in HBSS and stimulated with PMA (200 nM) for the indicated times. Chemiluminescence was assessed using the imaging system. (b) HT-29 cells were incubated in Opti-MEM and stimulated with PMA (200 nM) for the indicated times. Conditioned media was then used to measure extracellular ROS. HT-29 cells were pretreated with (c) NAC (10 mM) or (d) DPI (10 *μ*M) for 1 h prior to stimulation with PMA (200 nM) for 30 min. ROS concentration was calculated using a H_2_O_2_ standard curve (*n* = 3, ±SD). ^∗^ *p* < 0.01 and ^∗∗^ *p* < 0.001 vs. control.

Next, we tried to detect CL from PMA-treated cells using our chemiluminescent imaging system. By using different concentrations of H_2_O_2_ to create a reference, we found that 2 × 10^5^ HT-29 cells have 1.37 *μ*M (±0.24 *μ*M, S.D.) of basal ROS and PMA stimulation for 30 min increased ROS levels to 4.16 *μ*M (±0.13 *μ*M) (Figure 4(a)). As this method requires more cells than can fit inside a 96-well plate and the cells are kept in suspension, which can alter cellular signaling that requires integrin activation, we next tried to measure ROS from conditioned media from a 6-well plate as ROS freely diffuses out of the cell. As serum and phenol red may interfere with CL readings [27], we kept cells in phenol red-free Opti-MEM. Interestingly, we were able to detect ROS in conditioned media of PMA-treated HT-29 cells using our chemiluminescent imaging system (Figure 4(b)). Conditioned media from control cells had 1.35 *μ*M (±0.18 *μ*M) ROS in Opti-MEM (Figure 4(b)), similar to what we saw with cells in HBSS (Figure 4(a)). PMA increased the amount of extracellular ROS to 2.48 *μ*M (±0.40 *μ*M) in Opti-MEM (Figure 4(b)), which was lower than that of cells in HBSS (Figure 4(a)). Pretreatment with NAC or DPI blocked PMA-induced ROS production under both conditions, confirming that the CL signal is dependent on Nox1-induced ROS generation (Figures 4(c) and 4(d)). Together, these results suggest that the chemiluminescent imaging system is very useful for detecting and quantifying low levels of ROS and particularly total ROS generation in a real-time manner.

### 3.4. Effect of PMA-Mediated ROS Generation on Protein Tyrosine Kinase Activation

**Figure 5:**
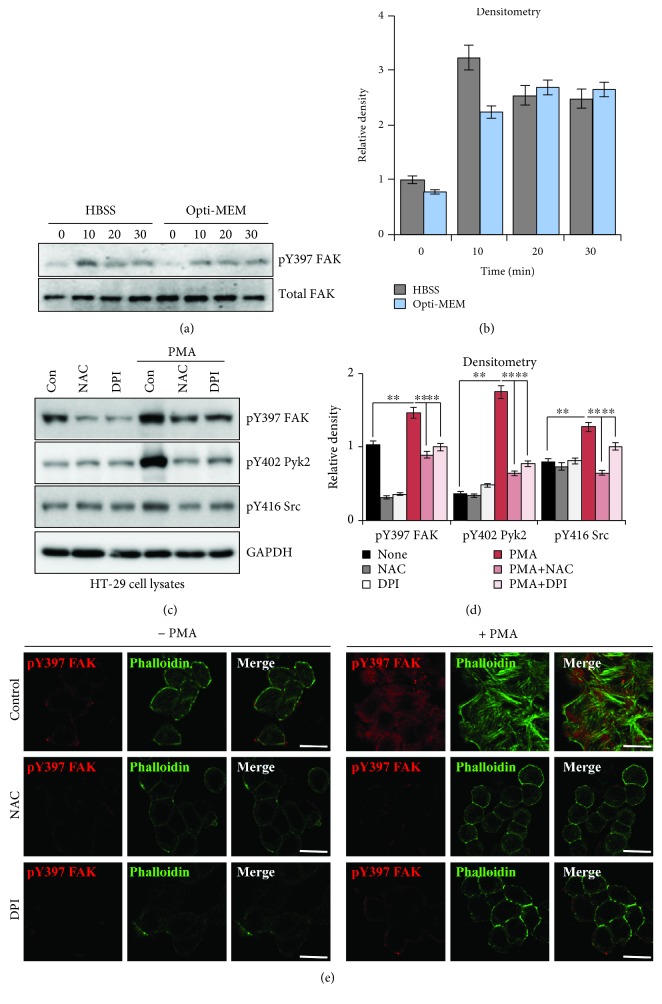
Effect of PMA-mediated ROS generation on tyrosine kinase activation. (a) HT-29 cells in 6-well plate were incubated in HBSS or Opti-MEM and treated with PMA (200 nM) for the indicated times. Shown are immunoblots of pY397 FAK and total FAK. (b) Fold change of pY397 FAK was calculated (*n* = 3, ±SD). (c–e) HT-29 cells were pretreated with NAC (10 mM) or DPI (10 *μ*M) for 1 h and then stimulated with PMA (200 nM) for 30 min. Cells were grown in 10% FBS DMEM. (c) Shown are immunoblots of pY397 FAK, pY402 Pyk2, pY416 Src, and GAPDH as the loading control. (d) Fold change of pY397 FAK, pY402 Pyk2, and pY416 Src was calculated (*n* = 3, ±SD). (e) Shown are immunostainings for pY397 FAK (red), actin stress fibers (green), and merge (red and green). Scale bar, 20 *μ*m (*n* = 4). ^∗∗^ *p* < 0.001 vs. control or vs. PMA.

The capability of measuring ROS in culture media allowed us to then use the cells for immunoblotting of downstream signaling molecules. Several PTKs, such as focal adhesion kinase (FAK), proline-rich tyrosine kinase 2 (Pyk2), and Src, have been shown to be activated by ROS [17–21, 28]. We investigated whether PMA-induced ROS generation could increase FAK activity in HT-29 cells. PMA increased FAK activity (western blotting monitored by pY397 FAK levels) from 10 min after PMA treatment and in three different medium conditions (HBSS, Opti-MEM, and DMEM) (Figure 5(a)–5(c)). In addition to FAK, PMA also increased Pyk2 and Src activity (monitored by pY402 Pyk2 and pY416 Src levels) (Figures 5(c) and 5(d)). NAC or DPI treatment reduced PMA-induced activation of FAK, Pyk2, and Src (Figures 5(c) and 5(d)), indicating that cellular ROS are a potent activator of PTKs. Interestingly, NAC or DPI treatment reduced basal levels of pY397 FAK, but not pY402 Pyk2 or pY416 Src (Figures 5(c) and 5(d)), implicating that FAK may have a potential role in ROS signaling even in a quiescent condition. Immunostaining revealed that pretreatment with NAC and DPI decreases basal levels of pY397 FAK and blocked PMA-induced FAK activation consistent with the western blot results (Figure 5(e)). Together, these data suggest that PMA-induced ROS indeed activate FAK in a Nox1-dependent manner in HT-29 cells.

Further, we found that while low dosage of H_2_O_2_ (10 *μ*M) increased pY397 FAK levels, higher concentrations (100 and 1000 *μ*M) decreased pY397 FAK levels (Supplemental Fig.  7). This suggests that varying amounts of ROS may have an opposing effect on cellular FAK activity. In addition, using a cell-free system, treatment with H_2_O_2_ increased pY397 FAK at all concentrations (Supplemental Fig.  6). These differences between intact cells and the cell-free system indicate that ROS may regulate pY397 FAK within the cell in a spatially organized manner.

## 4. Discussion

Chemiluminescence (CL) as a means to measure ROS was established long ago [8]. Because CL has high sensitivity, it is easy to use and more cost-effective than other ROS probes including fluorescence dyes, such as DCFDA, DCFH, and amplex red, and genetically encoded biosensors such as roGFP or cpYFP. While these recently developed methods and probes are useful, they still have their limitations including nonselectivity, nonspecific fluorescence, or requirement of transfection [7]. In this study, we established a new method for low-level ROS detection using both a luminometer and a chemiluminescent imaging system with a lower number of cells and within conditioned media, which offer more flexibility and quantification of the CL signal than traditional X-ray film-developing systems.

We have optimized a new technique for measuring low levels of ROS by using luminol dissolved in a NaOH- and 4IPBA-enhanced CL buffer and a chemiluminescent imaging system. This simple and rapid technique also provides strong linearity on varying H_2_O_2_ concentrations. Applying it to a high-throughput system may be useful for the development of therapeutics to ROS-related diseases.

Several studies suggested that an intracellular inhibitor of CL activity may be present in cells and that luminol may block release of ROS into the media [8, 29], thus limiting the amount of free luminol available to interact with ROS and HRP. To overcome this problem, we sought to look at enhancing the CL signal intensity. Compounds, such as coumaric acid, phenolic compounds, and phenylboronic acid derivatives, have been used to enhance the CL signal in luminol-based systems for the quantitative detection of proteins in western blotting [30, 31]. Among them, 4IPBA has been shown to have a very low background, increased signal intensity, and prolonged exposure time [13]. Here, we showed that 4IPBA could allow for the detection of low levels of ROS in cell-free and cell-based assays. The addition of 4IPBA increased the linear regression *R* ^2^ values in a time-dependent manner compared to luminol alone (Figure 1). Interestingly, 4IPBA increased the rate of the CL reaction, thus decreasing the duration of the reaction.

Luminol and lucigenin are prone to generate artifacts and often lead to false interpretations due mainly to redox cycling phenomena [32–34]. In particular, it is reported that CL reaction by using the luminol analog L-012 with HRP and H_2_O_2_ was inhibited by superoxide dismutase (SOD) and suggested this reaction could make superoxide. Thus, we also tested whether our CL buffer generated superoxide and could create a false signal. While the addition of SOD had no effect on the CL signal in our system, treatment with catalase (CAT), which targets H_2_O_2_, completely blocked the CL signal (Supplemental Fig.  7), suggesting that superoxide is not generated when using luminol, 4IPBA, HRP, and H_2_O_2_.

Using PMA, a strong ROS inducer in several cell types [25, 26], we found that 4IPBA increased the rate and intensity of the CL signal in HT-29 cells compared to luminol alone (Figure 3), potentially overcoming an inhibitory effect luminol may have on cellular ROS. 4IPBA addition peaked the CL signal at 8 min and had twice as much signal compared to luminol alone, which peaked at around 15 min (Figure 3). 4IPBA potentially doubles the rate at which luminol is oxidized and could more tightly reflect real-time ROS generation than luminol alone (Figure 3). A benefit to our system is that we can easily measure stimulus-induced ROS formation from media and interrogate intracellular signaling using a single sample because intracellular ROS readily diffuses out of the cell into the media (Figure 4). Also, H_2_O_2_ is comparatively stable compared to other ROS, such as superoxide, allowing for easy measurement of ROS within media [8, 27]. Thus, 4IPBA may enhance cellular condition for CL reaction as well as oxidation of luminol by HRP.

We also found that PMA-induced ROS formation correlated with pY397 FAK, pY402 Pyk2, and pY416 Src levels in HT-29 cells (Figure 5). Treatment with a ROS scavenger (NAC) or Nox inhibitor (DPI) prevented PMA-induced tyrosine phosphorylation of FAK, Pyk2, and Src, implicating that these PTKs can be indeed activated by cellular ROS (Figure 5(c)). However, basal levels of pY397 FAK, but not other PTKs, were reduced by NAC or DPI treatment, suggesting that low concentrations of ROS regulate FAK activity to maintain cellular homeostasis (Figure 5(c)). H_2_O_2_ is shown to inhibit some protein tyrosine phosphatases (PTPs) via oxidation (e.g., low molecular weight protein PTP for FAK), leading to increased activation of PTKs. Interestingly, while low levels of H_2_O_2_ increased pY397 FAK, higher dosages decreased pY397 FAK (Supplemental Fig.  6). These data suggest that high levels of ROS may cause cellular oxidative stress or may dephosphorylate pY397 FAK by interacting with FIP200 [35].

## 5. Conclusion

In summary, we have optimized a new technique for measuring low levels of ROS by using luminol dissolved in a NaOH- and 4IPBA-enhanced CL buffer and chemiluminescent imaging system. Not only does this technique provide simple linear results at an affordable cost, it can also be widely used for ROS-related research. Applying this new approach to a high-throughput system may be very useful for the development of an inhibitor or therapeutic agents to ROS-related diseases. Finally, we suggest that activation of PTKs, such as FAK, may be useful markers of stimulus-cellular ROS generation and downstream signaling.

## Figures and Tables

**Table 1:** Optimized chemiluminescence buffer composition to detect low levels of ROS.

Component^a^	Figure 1	Figure 2	Figure 3	Figure 4
Luminol^b^	25-200 *μ*M	50 *μ*M	50 *μ*M	50 *μ*M
4IPBA^c^	180 *μ*M	180 *μ*M	180 *μ*M	180 *μ*M
HRP^d^	0.1 or 0.2 units/ml	0.2 units/ml	0.2 units/ml	0.2 units/ml
H_2_O_2_^e^	1-10 *μ*M	1-10 *μ*M		—
PMA	—	—	200 nM	200 nM
Cells^f^	—	—	2 × 10^5^	2 × 10^5^ cells in HBSS
Supernatants	—	—	—	180 *μ*l from Opti-MEM (1.2 × 10^6^ cells)
Buffer^g^	HBSS	HBSS	HBSS	HBSS, Opti-MEM

^a^Final volume of the reaction was 200 *μ*l in a glass bottom 96-well plate. ^b^Luminol was dissolved in 100 mM NaOH or DMSO. Previous studies used 200 *μ*M or 1.25 mM luminol in DMSO [9, 13] or 50 *μ*M luminol dissolved in 100 mM NaOH [8]. ^c^4IPBA was dissolved in DMSO. A previous study used 200 *μ*M [13]. ^d^Previous studies used 0.32 unit/ml or 50 units of HRP [8, 9]- or HRP-conjugated antibody [13]. ^e^A previous study used 2.4 mM H_2_O_2_ [13]. ^f^Previous studies used 5 × 10^5^ or 10^2^-10^6^ cells [8, 9]. ^g^Previous studies used Hank's Balanced Salt Solution (HBSS), Krebs-Ringer bicarbonate buffer, or Tris buffer [8, 9, 13].

**Table 2:** Reagents and stock preparation for the optimized chemiluminescence buffer.

Component	Stock concentration	Storage	Working concentration
Luminol	50 mM, dissolved in 0.1 M NaOH or DMSO	Stored in small aliquots at -20°C	1 mM (diluted in HBSS)
4IPBA	90 mM, dissolved in DMSO	Stored in small aliquots at -20°C	3.6 mM (diluted in HBSS)
HRP	200 units/ml, dissolved in PBS	Stored in small aliquots at -20°C	4 units/ml (diluted in HBSS)
NAC	500 mM, dissolved in distilled water	Stored in small aliquots at -20°C	200 mM (diluted in HBSS)
DPI	1 mM, dissolved in DMSO	Stored in small aliquots at -20°C	200 *μ*M (diluted in HBSS)
SOD	1 mg/ml, dissolved in PBS	Stored in small aliquots at -20°C	1 mg/ml
CAT	30 KU/ml, dissolved in PBS	Stored in small aliquots at -20°C	30 KU/ml

## Data Availability

The data used to support the findings of this study are available from the corresponding author upon request.

## Abbreviations

CL: Chemiluminescence
CAT: Catalase
DPI: Diphenyleneiodonium
FAK: Focal adhesion kinase
HRP: Horseradish peroxidase
4IPBA: 4-Iodophenylboronic acid
NAC: N-acetyl-L-cysteine
Nox: NADPH oxidase
PMA: phorbol 12-myristate 13-acetate
PTK: Protein tyrosine kinase
PTP: Protein tyrosine phosphatase
ROS: Reactive oxygen species
SOD: Superoxide dismutase.

## Supplementary Materials

Supplementary Materials The supplemental information contains additional experimental methods, primer sequences, and supporting data. Supplemental Table 1: RT-qPCR primers. Supplemental Figure 1: complete kinetics profiles of the chemiluminescence assay. Supplemental Figure 2: kinetics of the chemiluminescence assay. Supplemental Figure 3: effect of 4IPBA on chemiluminescence. Supplemental Figure 4: effect of 4IPBA on HT-29 cell viability. Supplemental Figure 5: expression of Nox1 components in HT-29 cells. Supplemental Figure 6: effect of H_2_O_2_ on FAK activity. Supplemental Figure 7: effect of superoxide dismutase (SOD) or catalase (CAT) on chemiluminescence reaction.
