# Supplementary material for: A Quantitative Method to Measure Low Levels of ROS in Nonphagocytic Cells by Using a Chemiluminescent Imaging System
Source: Oxid Med Cell Longev. 2019 Jun 11;2019:1754593. doi: 10.1155/2019/1754593 (PMC6594271; doi:10.1155/2019/1754593)
Supplement: Supplementary Materials — The supplemental information contains additional experimental methods, primer sequences, and supporting data. Supplemental Table 1: RT-qPCR primers. Supplemental Figure 1: complete kinetics profiles of the chemiluminescence assay. Supplemental Figure 2: kinetics of the chemiluminescence assay. Supplemental Figure 3: effect of 4IPBA on chemiluminescence. Supplemental Figure 4: effect of 4IPBA on HT-29 cell viability. Supplemental Figure 5: expression of Nox1 components in HT-29 cells. Supplemental Figure 6: effect of H2O2 on FAK activity. Supplemental Figure 7: effect of superoxide dismutase (SOD) or catalase (CAT) on chemiluminescence reaction. [file 1754593.f1.pdf]

Supplemental Information

A quantitative method to measure low levels of ROS in non-phagocytic cells by using a chemiluminescent imaging system

Jun-Sub Kim[1,2], Kyuho Jeong[1], James M. Murphy[1], Yelitza A.R. Rodriguez[1], Ssang-Taek Steve Lim[1*]

Supplemental materials and methods

Chemiluminescence assay

Chemiluminescence (CL) was measured by luminol-amplified luminescence, and optimized CL buffer compositions are summarized in Table I. For optimization, H_2O_2 was mixed with luminol and HRP in 200 µl total volume in a glass bottom 96-well plate. For cells or conditioned media luminol, H_2O_2, and HRP were mixed in 200 µl total volume in a glass bottom 96-well plate. Luminescence was quantified by using a luminometer (Synergy Microplate Reader, BioTek).

Cell viability assay

The percentage cell viability was determined by the MTT [3-(4,5-dimethylthiazol-2-yl)-2,5-diphenyltetrazolium bromide] assay. HT-29 cells were seeded onto 96-well plates, maintaining a density of 2.0×10^4 cells per well in 200 µL culture medium and incubated for 24 h before addition of 4IBPA (0 - 1 mM) or 50 uM luminol (NaOH) + 4IBPA for another 2, 4, 24 h. This was followed by the addition of 20 µL MTT (0.5 mg/mL) to each well followed by incubation for 4 h at 37 °C. The culture medium was carefully aspirated and dissolved with 100 µL Dimethylsulfoxide (DMSO). This was allowed to stand for 10 mins. Optical density (OD) was read using an ELISA plate reader (BIO-RAD, USA) at absorption wavelength of 570 nm.

RNA extraction and quantitative real-time (RT) quantitative polymerase chain reaction

Total RNAs of HT-29 cells, HEK 293FT cells, human aortic endothelial cells (HAoECs), and human umbilical vein endothelial cells (HUVECs) were isolated using a kit (RNeasy kit, Qiagen) and converted to cDNA using random hexamers and reverse transcription (Superscript III, Life Technologies, Carlsbad, CA). RT quantitative polymerase chain reaction (RT-qPCR) was performed (iTaq Universal SYBR Green SMX and CFX connected optical module, Bio-Rad, Hercules, CA). All PCR reactions were performed with primers (Supplemental Table 1) with the following steps: initial denaturation, 95°C for 10 minutes; 40 cycles of denaturation, 95°C for 15 seconds and annealing/extension, 60°C for 60 seconds.

Immunoblotting

HT-29 cells were serum starved overnight and then treated with H_2O_2 for 30 min. Cells were lysed in 1% Triton X-100 lysis buffer. H_2O_2 was treated to lysates treated with 0.1% NP-40 Clarified lysates were run on 4-12% NuPage Tris-Bis gels (Life Technologies). Proteins transferred to PVDF membranes, blocked with 3% BSA and incubated overnight with primary antibodies at 4°C. Membranes were washed and incubated with HRP conjugated secondary, and proteins were then visualized using ECL on ChemiDoc MP Imaging System.

Supplemental Table 1. RT-qPCR primers

Gene Name	Sequence
**Nox1 (Forward)**	TGATCTGCCTACATACAGCTATTC
**Nox1 (Reverse)**	CATGAGATAGGCTGGAGAGAATG
**p22phox (Forward)**	GCCGTGGTGAAGCTGTT
**p22phox (Reverse)**	GCACCGAGAGCAGGAGAT
**NoxO1 (Forward)**	TTCTCGATGCACCACTGTT
**NoxO1 (Reverse)**	CAGCAGCCTCCGAGAATAG
**NoxA1 (Forward)**	AGCCTAAGGGAGGCCAT
**NoxA1 (Reverse)**	CCGTCTCTGCACTTGGTC

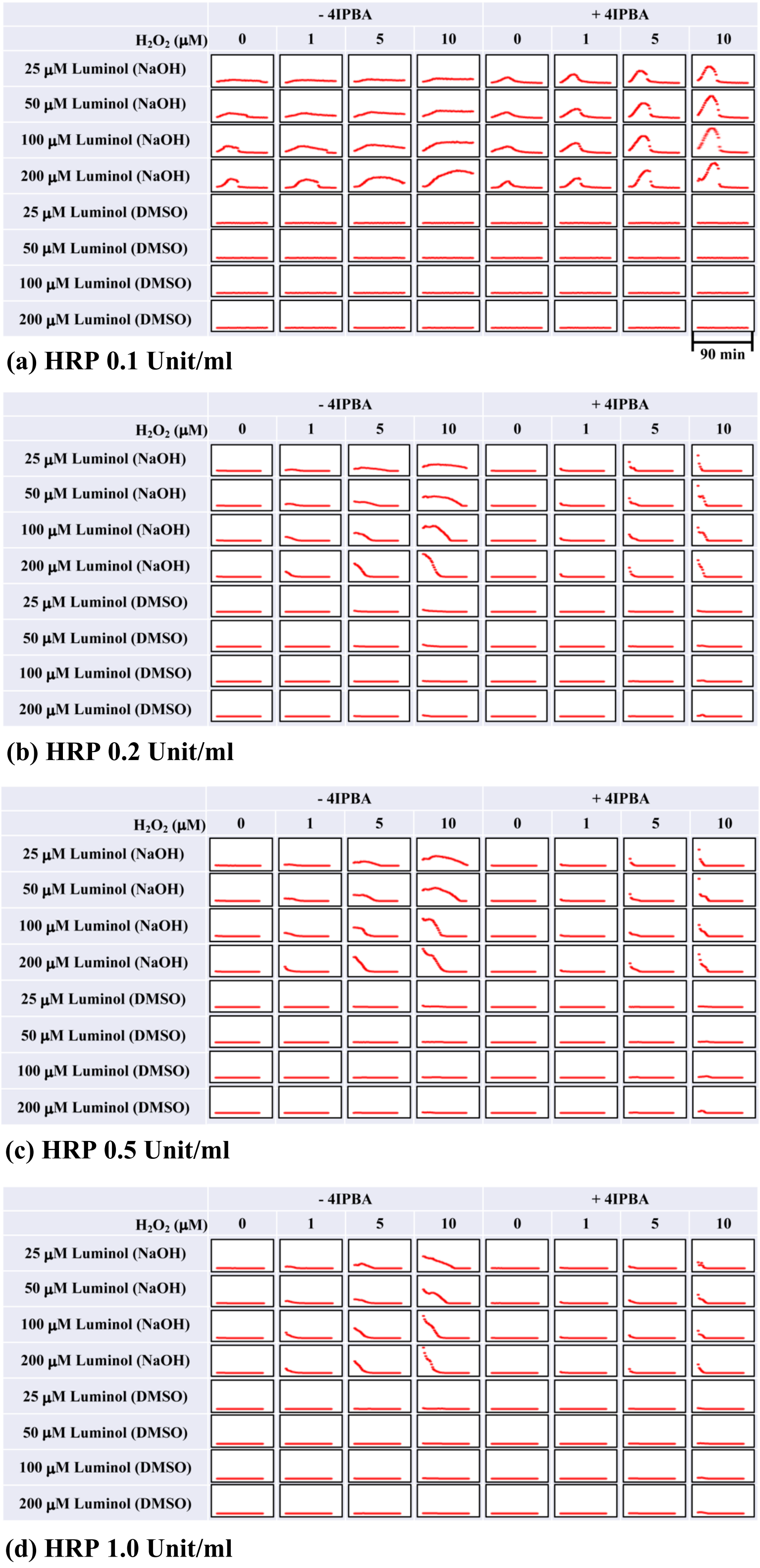

Supplemental Figure 1: Complete kinetics profiles of chemiluminescence assay
Chemiluminescence was measured over time using various concentrations of H_2O_2 (0, 1, 5, 10 µM), luminol (25, 50, 100, 200 µM) in DMSO or NaOH, 4IPBA (180 µM), and HRP (0.1, 0.2, 0.5, 1 unit/ml) in a black walled glass bottom 96-well plate. Chemiluminescence was measured using a luminometer for 90 min.

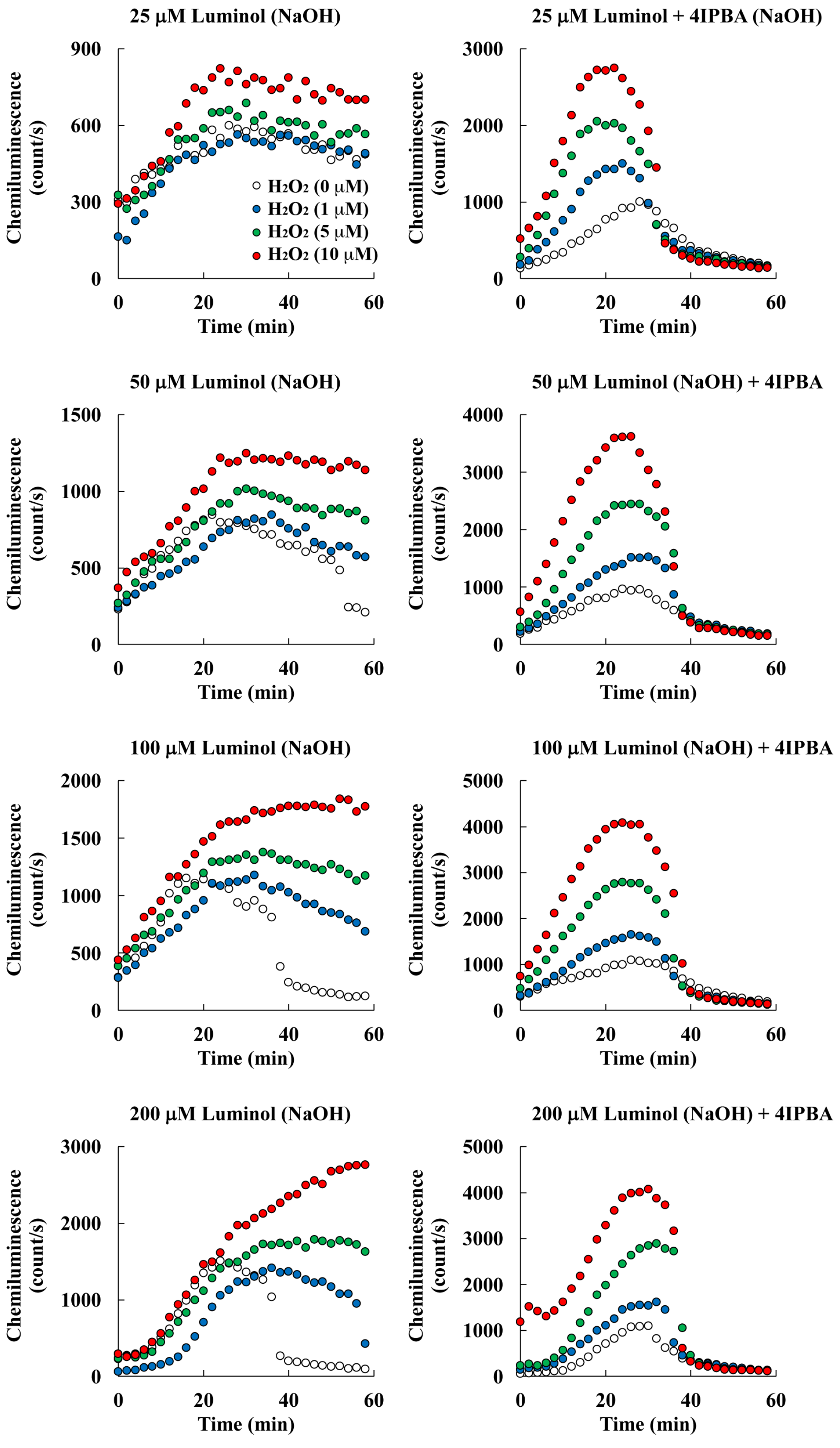

(a) HRP 0.1 Unit/ml

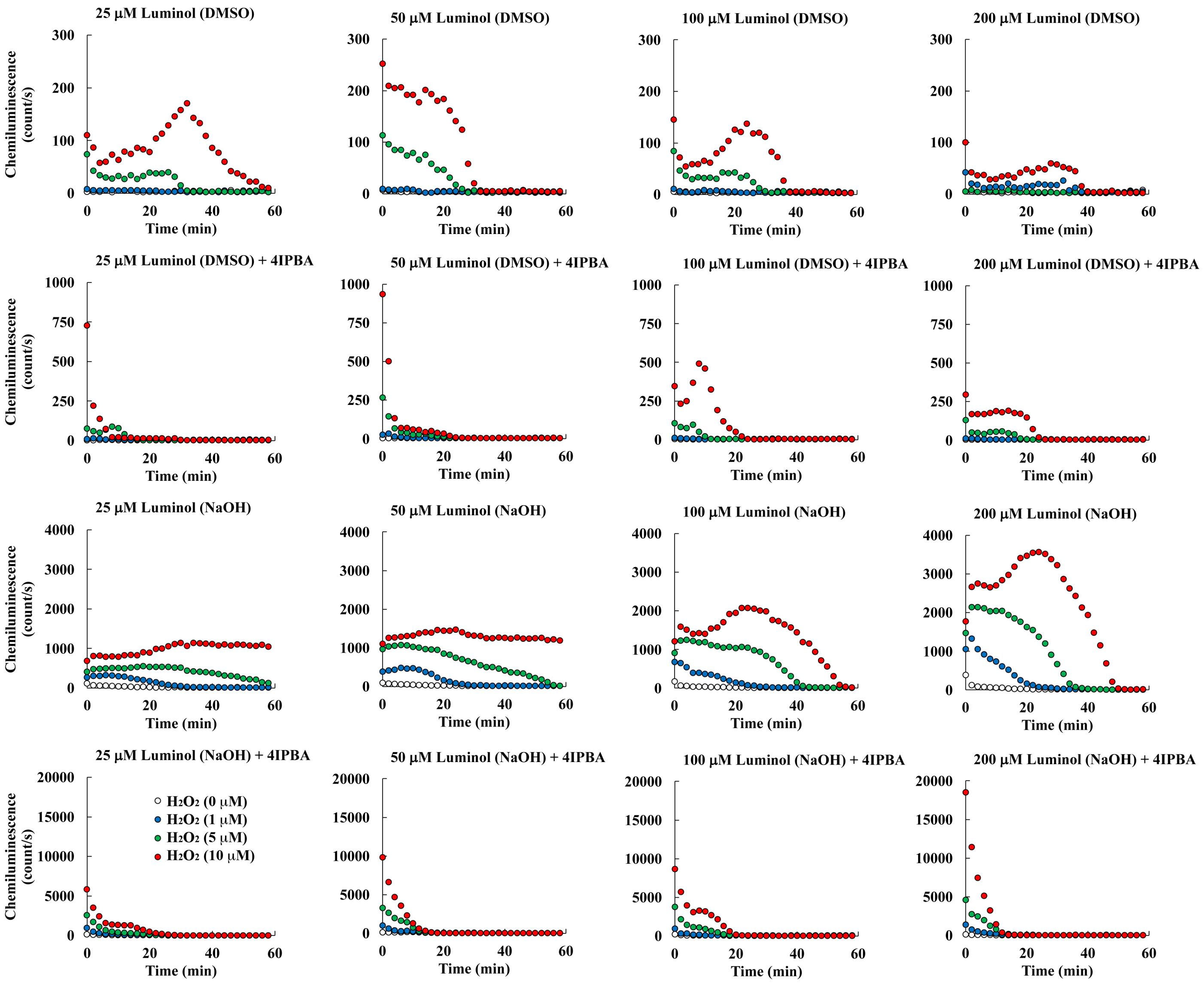

(b) HRP 0.2 Unit/ml

Supplemental Figure 2: Kinetics of chemiluminescence assay

Chemiluminescence was measured over time using various concentrations of H_2O_2 (0, 1, 5, 10 μM), luminol (25, 50, 100, 200 μM) in DMSO or NaOH, 4IPBA (180 μM), and HRP (0.1, 0.2 unit/ml) in a black walled glass bottom 96-well plate. Chemiluminescence was measured using a luminometer for 60 min (n=3).

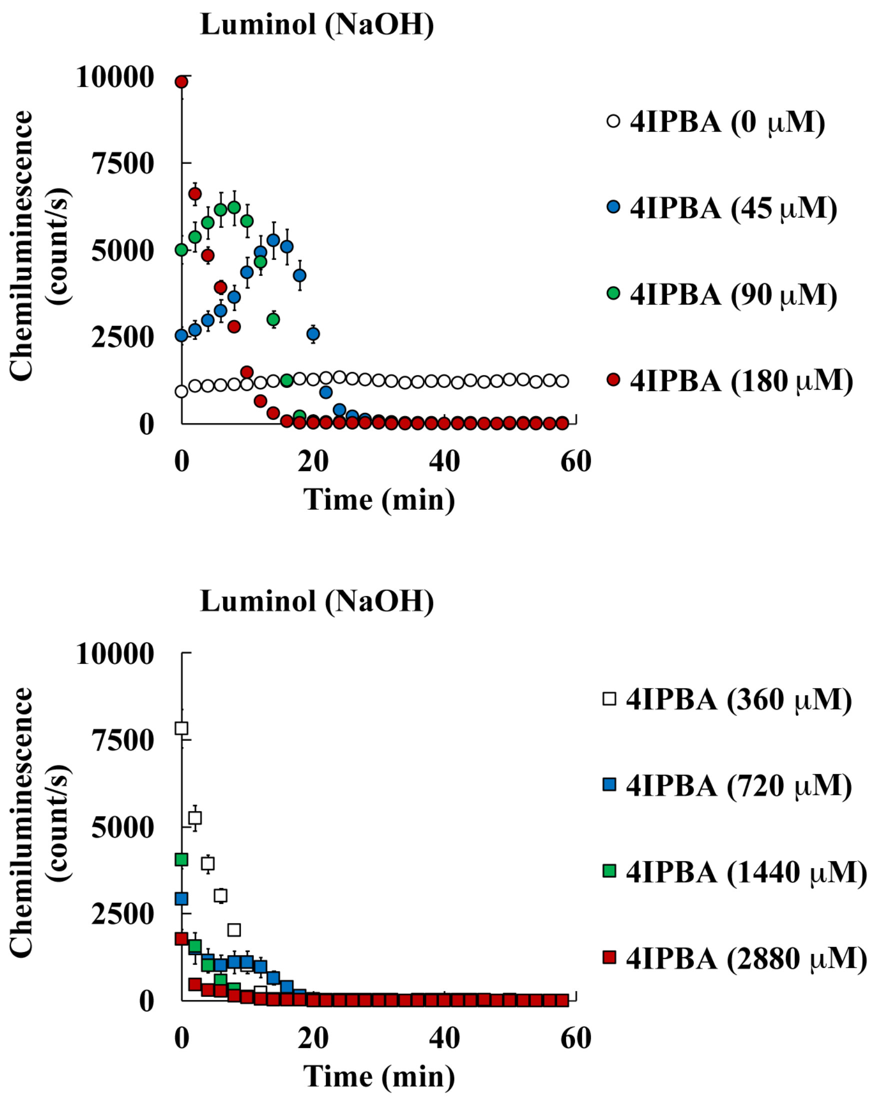

Supplemental Figure 3: Effect of 4IPBA on chemiluminescence.
Various concentrations of 4IPBA (0, 45, 90, 180, 360, 720, 1440, 2880 μM) were added to H_2O_2 (10 μM), luminol (50 μM in NaOH), and HRP (0.2 unit/ml) in a black walled glass bottom 96-well plate. Chemiluminescence was measured using a luminometer for 60 min (n=3, ±SD).

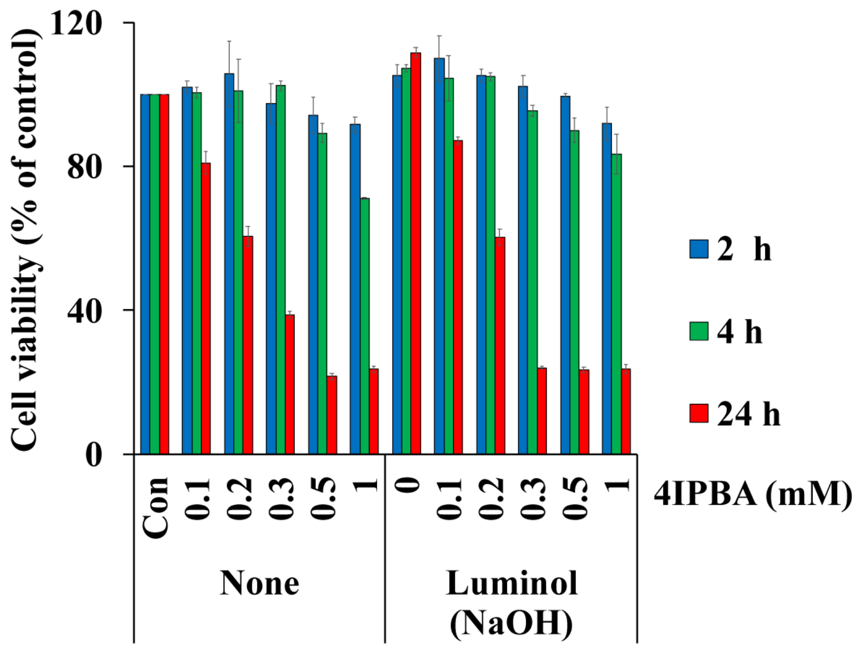

Supplemental Figure 4: Effect of 4IPBA on HT-29 cell viability

HT-29 cells were treated with increasing concentrations of 4IPBA (0, 0.1, 0.2, 03, 0.5, 1 mM) with or without luminol (50 µM in NaOH) for 2, 4, or 24 h. Cell viability were examined by MTT assay and calculated as percentage of control (n=3, ±SD).

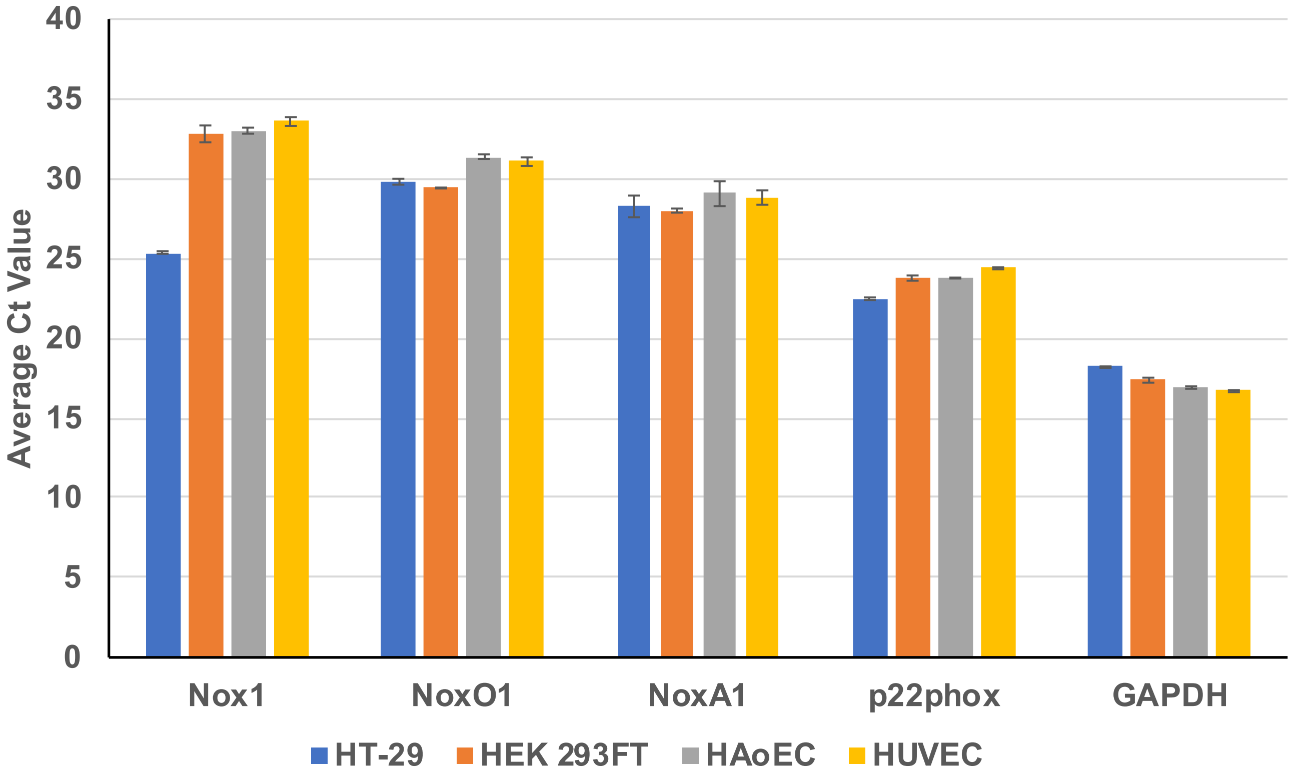

RT-qPCR Ct Values

	HT-29			HEK 293FT			HAoEC			HUVEC		
	1	2	3	1	2	3	1	2	3	1	2	3
Nox1	25.47	25.32	25.33	32.2	33.1	33.13	33.08	33.15	32.79	33.74	33.74	33.26
NoxO1	30.02	29.7	29.7	29.4	29.45	29.4	31.55	31.28	31.31	30.79	31.08	31.33
NoxA1	27.5	28.8	28.49	27.85	28.07	28.08	29.94	28.79	28.45	28.65	28.47	29.33
p22phox	22.51	22.54	22.38	23.7	23.65	23.96	23.79	23.74	23.79	24.34	24.36	24.46
GAPDH	18.21	18.13	18.23	17.54	17.34	17.23	16.84	16.89	17	16.66	16.64	16.76

Supplemental Figure 5: Expression of Nox1 components in HT-29 cells
Total RNAs from untreated basal HT-29 cells, HEK 293FT cells, human aortic endothelial cells (HAoEC), and human umbilical vein endothelial cells (HUVECs) were analyzed for Nox1 components (Nox1, NoxO1, NoxA1, and p22phox) by RT-qPCR. The bar graph represents average Ct value (n=3, ±SD). Table shows Raw Ct values.

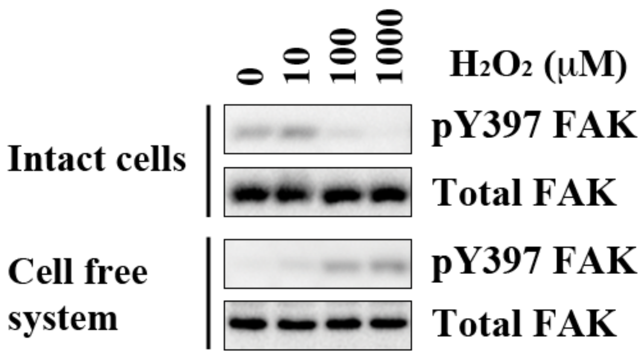

Supplemental Figure 6: Effect of H_2O_2 on FAK activity
Adherent HT-29 cells (intact cells) or HT-29 lysate (Tris buffer containing 0.1 % NP-40) were treated with increasing concentrations of H_2O_2 (0, 10, 100, 1000 μM) for 30 min. Samples were mixed 4X SDS sample buffer and immunoblotted for active FAK (pY397 FAK) or total FAK.

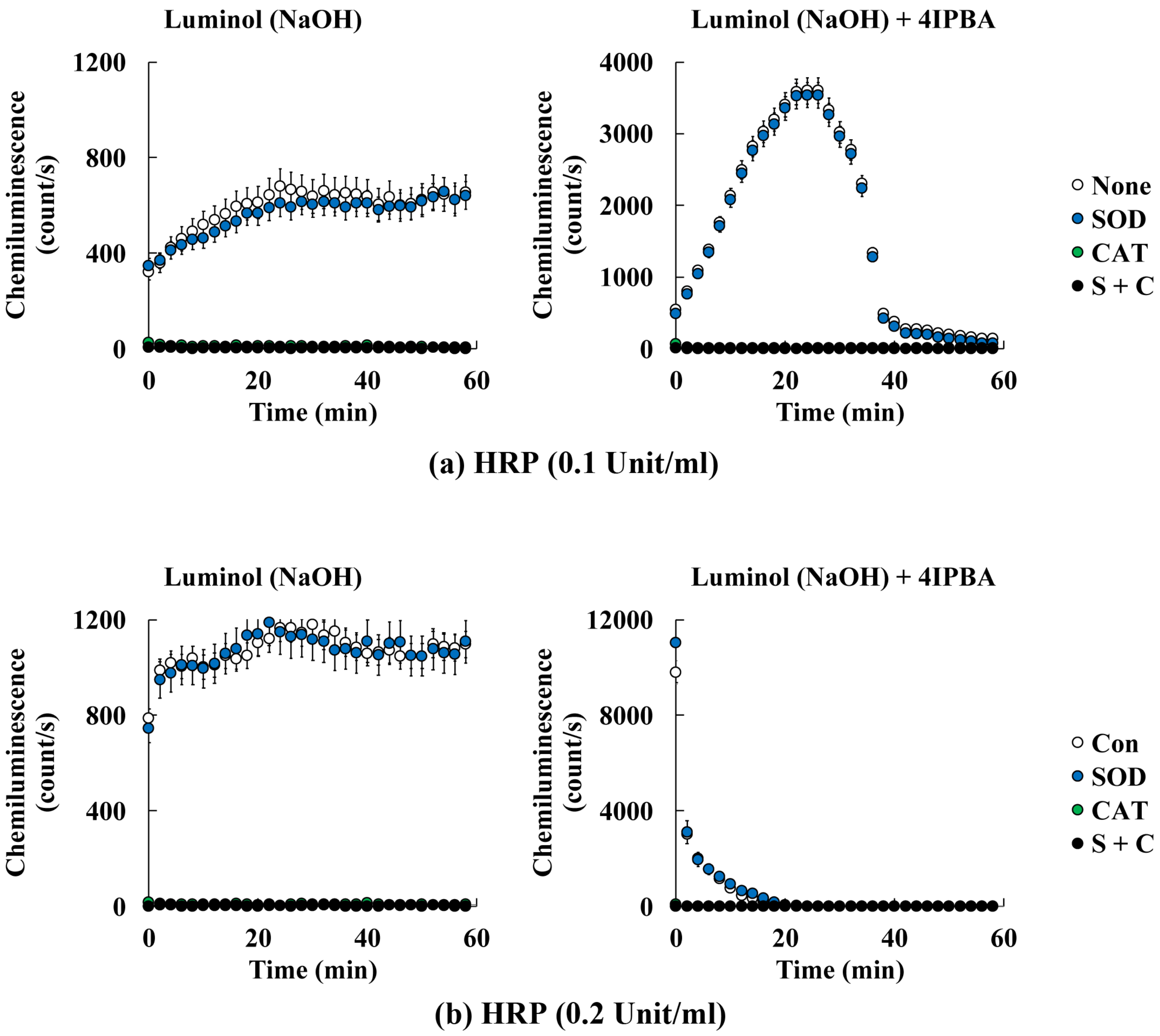

Supplemental Figure 7. Effect of superoxide dismutase (SOD) or catalase (CAT) on chemiluminescence reaction

H_2O_2 (10 μM) was mixed with luminol (50 μM in NaOH), 4IPBA (180 μM), and HRP (0.2 unit/ml) with or without SOD (50 μg/ml) and/or CAT (1.5 KU/ml) in a black walled glass bottom 96-well plate. Chemiluminescence was measured using a luminometer for 60 min (n=3, ±SD).
